# Effect of genotyped bulls with different numbers of phenotyped progenies on quantitative trait loci detection and genomic evaluation in a simulated cattle population

**DOI:** 10.1111/asj.13432

**Published:** 2020-08-11

**Authors:** Masayuki Takeda, Yoshinobu Uemoto, Masahiro Satoh

**Affiliations:** ^1^ National Livestock Breeding Center Nishigo Japan; ^2^ Graduate School of Agricultural Science Tohoku University Sendai Japan

**Keywords:** genomic evaluation, genotyped bulls, GWAS, simulated cattle population

## Abstract

The objective of this study was to assess the effect of genotyped bulls with different numbers of phenotyped progenies on quantitative trait loci (QTL) detection and genomic evaluation using a simulated cattle population. Twelve generations (G1–G12) were simulated from the base generation (G0). The recent population had different effective population sizes, heritability, and number of QTL. G0–G4 were used for pedigree information. A total of 300 genotyped bulls from G5–G10 were randomly selected. Their progenies were generated in G6–G11 with different numbers of progeny per bull. Scenarios were considered according to the number of progenies and whether the genotypes were possessed by the bulls or the progenies. A genome‐wide association study and genomic evaluation were performed with a single‐step genomic best linear unbiased prediction method to calculate the power of QTL detection and the genomic estimated breeding value (GEBV). We found that genotyped bulls could be available for QTL detection depending on conditions. Additionally, using a reference population, including genotyped bulls, which had more progeny phenotypes, enabled a more accurate prediction of GEBV. However, it is desirable to have more than 4,500 individuals consisting of both genotypes and phenotypes for practical genomic evaluation.

## 1 INTRODUCTION

A high‐density single nucleotide polymorphism (SNP) array has been widely utilized in various cattle breeds to detect quantitative trait loci (QTL) by genome‐wide association studies (GWAS) and to predict the genomic estimated breeding value (GEBV) by genomic evaluation. The power of QTL detection and the accuracy of GEBV are dependent on several factors, such as the genetic architecture of the traits (e.g. the number of QTL and heritability) and population size (e.g. the number of genotyped animals and effective population size; Daetwyler, Pong‐Wong, Villanueva, & Woolliams, 2010; Goddard & Hayes, 2007; Lourenco et al., 2017; Uemoto, Osawa, & Saburi, 2017). In particular, population size is a crucial factor, and a larger number of animals in the reference population is needed to increase the accuracy of GEBV in genomic evaluations (Daetwyler et al., 2010; Uemoto et al., 2017; VanRaden et al., 2009). However, high‐density SNP genotyping entails substantial costs to obtain a larger number of genotyped animals.

One of the strategies for cost‐effective genotyping is to utilize the genotypes of informative animals, such as progeny‐tested bulls, instead of the phenotyped animals. Progeny‐tested bulls have been used as a reference population in the genomic evaluation for Holstein populations in Japan because the progeny‐tested bulls have reliable information obtained from a larger number of their records for their daughters (Uemoto et al., 2017). Additionally, Takeda et al. (2020) reported that approximately 300 genotypes of progeny‐tested bulls with approximately 4,500 phenotypes of their progenies could have reliable information for QTL detection but might be insufficient for the accuracy of GEBV in Japanese Black cattle. This indicated that a sufficient power of QTL detection could be obtained by using progeny‐tested bulls even if a small number of genotyped animals is used in GWAS. However, there is no information regarding the effect of the number of phenotyped progenies on the power of QTL detection and the accuracy of GEBV when the progeny‐tested bulls with genotypes are utilized in a cattle population.

The objective of this study was to evaluate the effect of genotyped bulls with different numbers of phenotyped progenies on the power of QTL detection and the accuracy of GEBV by using a simulated cattle population. In the simulation analysis, 300 genotyped bulls with no phenotype but with different numbers of phenotyped progenies were regarded as the population of GWAS and the reference population for genomic evaluation. Additionally, the different number of progenies with both genotype and phenotype were used for comparison with the results of genotyped bulls. The power of QTL detection and accuracy of GEBV using these populations were evaluated under varying conditions, including different effective population size (Ne), heritability (h^2^), and number of QTL (nQTL).

## 2 MATERIALS AND METHODS

Animal Care and Use Committee approval was not needed because data were simulated.

### 2.1 Simulated population

**FIGURE 1:**
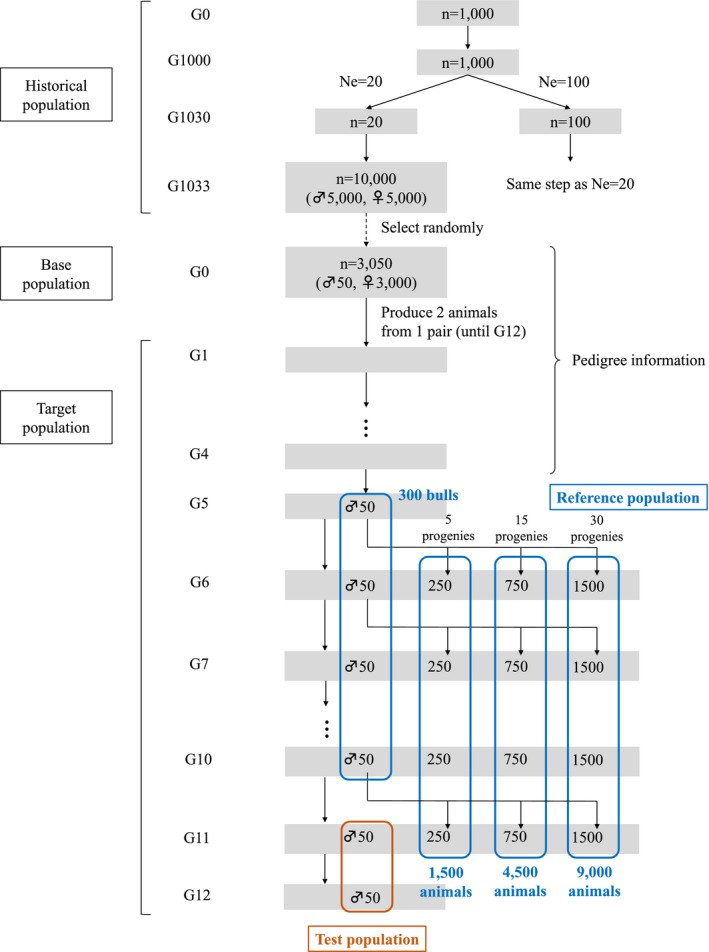
Schematic illustration of the simulation process

Populations were simulated based on the forward‐in‐time process (Carvajal‐Rodríguez, 2008) using QMSim software (Sargolzaei & Schenkel, 2009). The schematic illustration of the simulation process is shown in Figure 1. A historical population was simulated to create a mutation‐drift equilibrium and linkage disequilibrium (LD). The size of the historical population began with 1,000 individuals and was generated as generation 0 to 1,000, with a constant size of 1,000. Two different populations with different Ne were generated with a Ne of 20 and 100, which mimicked the recent Ne of Japanese Black cattle populations (Nomura, Honda, & Mukai, 2001) and other cattle breed populations (Lourenco et al., 2017), respectively. Thus, the number of individuals was gradually reduced from 1,000 to 20 (for the Ne of 20) or 100 (for the Ne of 100) from generation 1,001–1,030, respectively. It was then expanded to 10,000 after three generations, resulting in 5,000 males and 5,000 females in the last generation (i.e., generation 1,033) of the historical population. In each population, 50 males and 3,000 females were randomly selected from the last historical generation to be founders for the recent population.

The recent population was simulated for 12 non‐overlapping generations (G1 to G12). Each mating produced two progenies with equal chances of being male or female, and 50 male and 3,000 female progenies were then randomly selected for mating in each generation. The base population (G0) and the first four generations (G1–G4) were used only for pedigree information in the later analyses. Fifty bulls were randomly selected in each generation from G5 to G10. Five, 15, and 30 progenies with phenotypic data per the bull from G6 to G11 were also randomly selected. These phenotyped progenies were not selected as the sire or dam of the next generation. In total, 300 non‐phenotyped bulls from G5 to G10 and different numbers of their phenotyped progenies (1,500, 4,500, and 9,000 progenies) from G6 to G11 were used in the validation study.

For the population and genome structures, the recent population had different conditions with the following varying factors: Ne, h^2^, and nQTL. The simulated genome consisted of 30 autosomal chromosomes with a length of 100 cM and randomly located bi‐allelic SNPs (*n* = 3,000) and QTL (*n* = 50) in each chromosome. Then, a total of 50,000 SNPs and different nQTL (10, 50, and 500) with minor allele frequencies >0.05 were randomly selected from all chromosomes (total length of 3,000 cM and 30 chromosomes). The SNP mutation rate and QTL mutation rate were set at 2.5 × 10^–5^. The QTL effects were sampled from a gamma distribution with a shape parameter of 0.4 and a scale parameter determined internally for the simulated genetic variance. The simulated phenotypes with the set value of phenotypic variance (1.0) were generated with two set values of h^2^ (0.20 and 0.50) explained only by simulated QTL. These conditions in each factor are summarized in Table 1. A total of 10 replicates of historical and recent populations were simulated for each condition.

**TABLE 1:** Factors for different conditions in a simulated population

Factors	Condition
Effective population size (Ne)	20, 100
Number of QTL (nQTL)	10, 50, 500
Heritability (h^2^)	0.20, 0.50

### 2.2 Statistical models using single‐step genomic BLUP (ssGBLUP)

The ssGBLUP approach proposed by Aguilar et al. (2010) and Wang, Misztal, Aguilar, Legarra, and Muir (2012) was applied to perform GWAS and genomic evaluation. The GWAS and genomic evaluation were performed using the BLUPF90 family of programs (Aguilar et al., 2018). The detailed description of the statistical models used is in Takeda et al. (2020), and a brief description is as follows. The single‐trait animal model was used for the genomic analyses as follows:

(1) $$y=1_{n}\mu +\mathrm{Zu}+e$$

where **y** is the vector of simulated phenotype; **1**_n_ is a vector of *n* ones; *μ* is the mean; **Z** is the design matrices for **u**; **u** and **e** are the vectors of GEBVs with $u∼N\left(0,\;H\sigma_{u}^{2}\right)$ and random error effect with $e∼N\left(0,\;I\sigma_{e}^{2}\right)$, respectively, where $\sigma_{u}^{2}$ and $\sigma_{e}^{2}$ are additive genetic and error variances, respectively. **I** is an identity matrix and the inverse of matrix **H** is calculated as follows:

$$H^{-1}=A^{-1}+\left[\begin{array}{cc} 0 & 0\\ 0 & {\left(\alpha G+\beta A_{22}\right)}^{-1}-A_{22}^{-1} \end{array}\right]$$

where **A** is the additive relationship matrix (ARM); **A**_22_ is the ARM for genotyped animals; *α* and *β* were weighting factors; **G** is the genomic relationship matrix (GRM) proposed by VanRaden (2008). The GRM was adjusted to be on the same scale of allele frequency in the base population using the method of Christensen (2012); this is the default setting for the BLUPF90 family of programs (Aguilar et al., 2018).

For GWAS, the weighting factors in $H^{‐1}$ (*α* and *β*) were selected as 1 and 0, respectively, and variance components were estimated using model (1). The GEBVs were then predicted and the estimated SNP effects ($\hat{\beta }$) were obtained using the following equation:

$$\hat{\beta }={\mathrm{DW}}^{\prime }{\left({\mathrm{WDW}}^{\prime }\right)}^{-1}{\hat{u}}_{g}$$

where **W** is a matrix relating to genotypes for each locus; **D** is a diagonal matrix of weights for variances of SNP (initially **D** = **I**); and ${\hat{u}}_{g}$ is a vector of GEBV of genotyped animals. The procedure, which consists of a GEBV computation and the refinement of SNP weights through two iterations, was performed to estimate the SNP effect as described by Wang et al. (2012). The proportion of genetic variance explained by the *i*‐th region was calculated by a window of 20 adjacent SNPs. For genomic evaluation, the phenotypic variance was 1.0 and the weighting factors in $H^{‐1}$ (*α* and *β*) were selected as 0.95 and 0.05, respectively, which were the default values. The estimated breeding values (EBVs) were also predicted by using model (1) with the same variance components of prediction for GEBV, but **u** was replaced as the vector of random effects because of EBV with $u∼N\left(0,\;A\sigma_{u}^{2}\right)$.

### 2.3 Scenarios and validation

For the population of GWAS and the reference population of genomic evaluation, the 300 genotyped bulls with no phenotype but with three different numbers of phenotyped progenies (9,000, 4,500, and 1,500) were used in this study. Additionally, the same number of progenies with both genotype and phenotype (9,000, 4,500, and 1,500), but no genotypes for their sires were also used for comparison with the results of the genotyped bulls. The seven different scenarios (SCEN0‐6) were set and summarized in Table 2 as follows: SCEN1–3 had 9,000, 4,500, and 1,500 progenies with both phenotypic and genotypic data, respectively, and SCEN4–6 had 9,000, 4,500, and 1,500 progenies with only phenotypic data and genotypic data for their sires (300 bulls), respectively. The genotypic data were composed of SNP data (i.e., 50,000 SNPs). SCEN0 represented as a true value, which had the same composition as that of SCEN1 but had information of both SNP and QTL positions.

**TABLE 2:** The reference population dataset for the 10 scenarios

Scenario^a^	Bull (*N* = 300, G5‐10)	Progenies (G6‐11)		
	Genotype	*N*	Genotype	Phenotype
SCEN0	×	9,000	〇	〇
SCEN1	×	9,000	〇	〇
SCEN2	×	4,500	〇	〇
SCEN3	×	1,500	〇	〇
SCEN4	〇	9,000	×	〇
SCEN5	〇	4,500	×	〇
SCEN6	〇	1,500	×	〇
SCEN7	×	9,000	×	〇
SCEN8	×	4,500	×	〇
SCEN9	×	1,500	×	〇

^a^ Genotyped animals have both single nucleotide polymorphism (SNP) and QTL positions in SCEN0, and they have only SNP data in SCEN1‐9.

For the power of QTL detection, GWAS was performed in SCEN0 first, and the QTL explaining more than 1.0% of the genetic variance were obtained. The threshold was chosen based on a previous weighted single‐step GWAS (Marques et al., 2018; Takeda et al., 2020), which reported rationale results. Among all the detected QTL, the QTL on the set true QTL positions were regarded as a detectable QTL (trueQTL). GWAS with other scenarios (SCEN1–6) were then performed, and the number of detected QTL with 1.0% of the genetic variance on trueQTL (n_detQTL) was counted. The power to detect QTL was defined as the proportion of n_detQTL relative to the number of trueQTL. For the accuracy of GEBVs in genomic evaluation, 50 bulls that had SNP data and true breeding values (TBVs) were randomly selected in each generation from G11 to G12. These 100 bulls were regarded as the test population. The accuracy of GEBV was calculated as the correlation coefficient between TBV and GEBV in all scenarios (SCEN0–6). Additionally, the accuracy of EBV was also calculated to compare with those of GEBV according to the scenarios (SCEN7–9) in Table 2, which had 9,000, 4,500, and 1,500 progenies with phenotypic data, respectively. In SCEN7–9, no animal had genotypic data. The mean and standard deviation (*SD*) of 10 replicates was calculated in each scenario under different conditions.

## 3 RESULTS

### 3.1 Linkage disequilibrium

**FIGURE 2:**
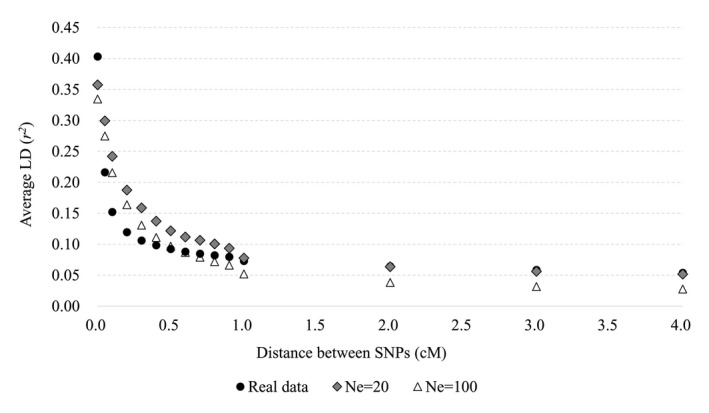
Average linkage disequilibrium coefficient values (*r* ^2^ values) plotted against intermarker distance for all autosomal chromosomes for real and simulated data (Ne = 20 and 100). The *x*‐axis indicates the distance between single nucleotide polymorphisms (SNPs) and the *y*‐axis indicates the *r* ^2^ values between SNPs

The pattern of LD decay of the real and simulated population was compared to evaluate the adequacy of the simulation process. We obtained *r* ^2^ values that are measures of LD between any two loci in the base population and are supplied by a feature of QMSim software (Sargolzaei & Schenkel, 2009). The *r* ^2^ values in two simulated populations (Ne = 20 or 100) were randomly extracted and are shown in Figure 2. The simulated population with Ne = 20 had higher *r* ^2^ values than did that with Ne = 100 in all distances between two loci. Additionally, the *r* ^2^ values, which were obtained from 547,043 SNP genotypes of 362 Japanese Black bulls as reported by Takeda et al. (2020), are also shown in Figure 2 to compare with those of the two simulated populations. To compare the difference in the *r* ^2^ values between the real data of Japanese Black bulls and the simulated populations, the scale of intermarkder distance (Mbp) in the real data was assumed as cM. The *r* ^2^ values of the two simulated populations did not have the same value as that of the real data in less than 1 cM distance between two loci. However, the *r* ^2^ values of the simulated population with Ne = 20 had similar values to that of the actual data in more than 1 cM distance between two loci.

### 3.2 GWAS

**FIGURE 3:**
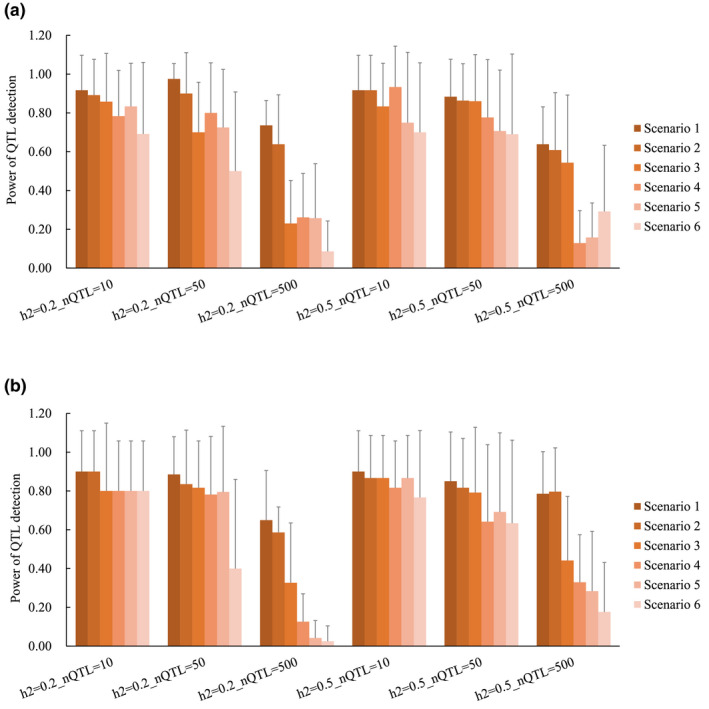
The power of quantitative trait loci (QTL) detection in the scenarios. (a), Ne of 20; (b), Ne of 100; h^2^, heritability; nQTL, number of QTL. The y‐axis indicates the power of QTL detection

The power of QTL detection in SCEN1–6 is shown in Figure 3. Among SCEN4–6, which were considered genotyped bulls with a different number of phenotyped progenies, the power of QTL detection was generally higher with the increasing number of progeny per bull. In the population with Ne = 20, for example, had detection powers in SCEN4, SCEN5, and SCEN6 that ranged 0.13–0.93, 0.16–0.83, and 0.09–0.70, respectively. When the nQTL was 10 or 50, the detection powers were moderate to high (ranging from 0.50 to 0.93 in the population with Ne = 20 and ranging from 0.40 to 0.87 in the population with Ne = 100). On the other hand, in the case of 500 QTL, the detection powers were low (ranging from 0.09 to 0.29 in the population with Ne = 20 and ranging from 0.03 to 0.33 in the population with Ne = 100). The h^2^ and Ne did not affect the power of QTL detection among SCEN4–6.

For SCEN1–3, which the progenies had both genotypes and phenotypes, the powers of QTL detection in SCEN1–3 were generally higher than those in SCEN4–6. Additionally, the detection powers were higher with an increasing number of animals with both phenotypes and genotypes. For example, when Ne = 20, h^2^ = 0.2, and nQTL = 50, the highest detection power of 0.98 occurred in SCEN1, followed by those of 0.90 and 0.80 in SCEN2 and SCEN3, respectively. The detection powers for nQTL = 500 were lower than those for nQTL = 10 or 50, but the degrees were not as remarkable as those in SCEN4–6. For example, when the Ne was 100 and the h^2^ was 0.2, the detection power in SCEN1 for nQTL = 500 was 0.65, but that in SCEN4 was 0.13. Comparing between SCEN1–3 and SCEN4–6, there were differences in the detection power when nQTL was 500, but no significant differences for nQTL = 10 or 50 were observed. The h^2^ and Ne did not affect the detection power between SCEN1–3 and SCEN4–6.

### 3.3 Genomic evaluation

**FIGURE 4:**
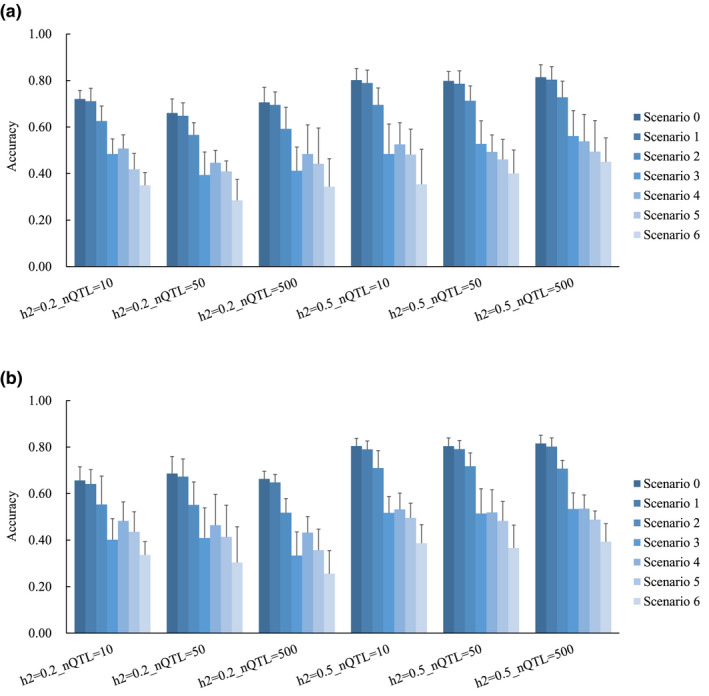
The accuracy of genomic estimated breeding values in the scenarios. (a), Ne of 20; (b), Ne of 100; h^2^, heritability; QTL, quantitative trait loci; nQTL, number of QTL. The *y*‐axis indicates the accuracy of genomic estimated breeding values

The accuracies of GEBVs in SCEN0–6 are shown in Figure 4. For comparison among SCEN4–6, a consistent trend was observed in that the higher accuracy of GEBV was obtained with a larger number of progeny per bull. The accuracies for SCEN4–6 in the population with Ne = 20 and Ne = 100 ranged 0.45–0.54 and 0.43–0.54, respectively. The nQTL and Ne did not affect accuracy. On the other hand, the accuracy increased with heritability.

The accuracies in SCEN0 were the highest among the seven scenarios and ranged 0.66–0.81 and 0.66–0.82 in the population with Ne = 20 and Ne = 100, respectively. They were almost the same as those in SCEN1, which ranged 0.65–0.80 and 0.64–0.80 in the population with Ne = 20 and Ne = 100, respectively. Comparing among SCEN1–3, the larger number of animals with both phenotypes and genotypes led to higher accuracies. Even in SCEN1–3, there was no effect of nQTL and Ne. The accuracies in SCEN1 and SCEN2 were generally higher than those in SCEN4–6. In many cases, the results of SCEN3 were slightly lower than those in SCEN4 and were similar to those of SCEN5. For example, when the Ne was 20 and h^2^ was 0.2, the accuracies in SCEN3 ranged 0.39–0.48 and were lower than those in SCEN4, ranging 0.45–0.51. The nQTL and Ne did not affect the power of the accuracy of GEBV between SCEN1–3 and SCEN4–6. The accuracies of EBVs in SCEN7–9 are shown in Figure S1. Overall, the accuracies were <0.1.

## 4 DISCUSSION

The size of the reference population is known as an important factor for the power of QTL detection and prediction accuracy of GEBV. However, the cost of genotyping is still a big issue. Because progeny‐tested bulls have progenies with records, genotypes of the bulls may be informative for genetic analysis. Therefore, we evaluated the availability for genotyped bulls on GWAS and genomic evaluation. The quality of information for bull genotypes is dependent on the number of progenies per bull. Accordingly, we generated different numbers of phenotyped progenies in bulls and examined the power of QTL detection and accuracy of GEBV, using genotypes of the bulls and phenotypes of the progenies. From the results, the following showed the validity of the simulated population, with a discussion for GWAS and genomic evaluation.

In this study, two different Ne were considered because Japanese Black beef cattle (Ne = 20) and Holstein dairy cattle (Ne = 100) are the major breeds in Japan. Almost the same LD patterns between the real and simulated populations with Ne = 20 were observed in more than 1 cM distance between two loci. Hence, the LD structure of the simulated population with Ne = 20 seems to be similar to that of the real data. On the other hand, the LD patterns of two simulated populations were not the same as that of the real data with less than 1 cM distance between two loci. One of the reasons is that the real data had SNP genotypes based on an imputed BovineHD SNP array (Takeda et al., 2020), whereas the simulated data had SNP genotypes based on a 50 K SNP array. The LD pattern in a narrow region could be estimated more accurately by utilizing the closely located SNP information. Regarding the power of QTL detection and the accuracy of GEBV in the two populations (Ne = 20 and 100), no large difference was observed in both results. In general, Ne has a close relationship with the extent of LD, and thus, a lower Ne can lead to a higher LD. Because the LD is higher, the superior ability to detect true QTL was observed (Melo et al., 2016). On the other hand, it is known that the extent of the LD affects the accuracy of GEBV (Garrick, 2010; Goddard, Hayes, & Meuwissen, 2010; Taylor et al., 2009). For these reasons, it was expected that some impact because of LD would be observed. However, as mentioned below, the Ne and LD did not contribute to the power of QTL detection and the accuracy of GEBV in the current simulated populations. One of the reasons might be that the value of Ne was different, but the same *Bos taurus* species as Japanese Black and Holstein breeds were assumed. In the simulation study of Melo et al. (2016), the higher and lower LD populations mimicked the LD of two different species of *Bos taurus* and *Bos indicus*. On the contrary, Goddard (2009) proposed a formula to determine the reliability of GEBV using Ne, number of individuals with phenotypic records (N), h^2^, and the length of chromosome (L), although the number of markers was not considered. In order to validate our results, we determined the accuracy of GEBV considering the square root of reliability depending on the Ne (20, 100, 250, 500, or 1,000) using the conditions of our study (i.e., *N* = 9,000, 4,500, or 1,500; h^2^ = 0.5 or 0.2; and L = 30). The results are presented in Figure S2. We found that the calculated accuracies did not substantially change with the increase in Ne, suggesting that the contribution of Ne to the accuracy of GEBV is small under the present study conditions. This further supports our results.

For GWAS, previous simulation studies in livestock have reported the effects of various factors on the power of QTL detection, such as nQTL, h^2^ (Van den Berg, Fritz, & Boichard, 2013), phenotypic information of non‐genotyped animals, and statistical methods (Melo et al., 2016). The current study showed that the power of QTL detection was low for polygenic traits, agreeing with Van der Berg et al. (2013) who performed QTL mapping by estimating the SNP effects using Bayesian methods. They also reported that higher h^2^ increased the accuracy of QTL detection. This is inconsistent with the current study where h^2^ did not have a large effect. Our study evaluated the effects of the genotyped bulls with a different number of progenies, which has not yet been investigated. The results showed that a smaller number of progeny per bull led to the lower power of QTL detection. When using both genotypes and phenotypes of the progenies, higher powers of QTL detection were observed. For traits with nQTL = 10 or 50, the powers of QTL detection in the case of bull genotype use were not lower than those using both genotypes and phenotypes of the progenies. However, for the traits with nQTL = 500, the powers of QTL detection were low. This can be because the proportion of each QTL variance in the total genetic variance is small when the QTL effects were determined from gamma distribution; thus, most QTL effects were small. On the contrary, for the traits with nQTL = 10 or 50, the proportion of each QTL variance in the total genetic variance is large, although the QTL effect was small. Hence, the influence of QTL effect will be larger than that of polygenic traits. These results indicated that using the genotype of progeny‐tested bulls is valuable when the bulls have a large number of progenies and the target traits are less polygenic. Additionally, the approach is cost‐effective for genotyping. However, it should be noted that using a population with a small Ne can lead to an increase in the length of LD, and thus, it will be difficult to perform QTL fine mapping.

For genomic evaluation, the accuracies of EBVs among the scenarios (SCEN7–9) were low and cannot be compared to each other because of the large standard deviations. In contrast, apparent differences in the accuracies of GEBVs among scenarios (SCEN1–6) were observed. In our study, the influence of h^2^ seemed to be larger than that of nQTL and Ne. This is consistent with the results of Brito, Neto, Sargolzaei, Cobuci, and Schenkel (2011) and Piccoli et al. (2017). Brito et al. (2011) reported that the accuracy of GEBV increased significantly with an increase in h^2^ from 0.1 to 0.4, using a simulated beef cattle population. Piccoli et al. (2017) estimated h^2^ for economic traits, which ranged from 0.10 to 0.46 in Brazilian Bradford and Hereford cattle, and showed a higher accuracy of GEBV with higher h^2^. Under any condition, the number of animals in the reference population had a large effect on the accuracy of GEBV in our study. This is in agreement with previous simulation studies (Brito et al., 2011; Lourenco et al., 2017).

In this study, the simulated populations with non‐overlapping generation were assumed because it is easy to understand the effects of genotyped bulls with different numbers of phenotyped progenies on QTL detection and genomic evaluation and compare with the results of Takeda et al. (2020). However, in a real‐world breeding program of Japanese Black cattle, the elite bulls, which have prominent marbling, have been utilized multiple times across generations (Nomura et al., 2001). The use of these elite bulls with overlapping generations in the genomic study might affect the accuracy of GEBV in genomic evaluation. When these elite bulls are included in the reference population, the genomic relationship between the reference and test populations might become closer, and thus, the reliability of GEBV for the test population would increase (Habier, Tetens, Seefried, Lichtner, & Thaller, 2010; Pszczola, Strabel, Mulder, & Calus, 2012; Wu, Lund, Sun, Zhang, & Su, 2015). Therefore, the accuracy of GEBV in a real cattle population might be higher than that obtained in our study.

As the Ne of Japanese Black cattle is small (Nomura et al., 2001), it is difficult to collect sufficient genotype data for progeny‐tested bulls. Accordingly, the number of progeny‐tested bulls was assumed to be a practical value of 300 in this study. The structure of the reference population in SCEN5 was similar to our previous study (Takeda et al., 2020), which included 3,773 progenies with phenotypes and the 295 bulls with genotypes. For the power of QTL detection, Takeda et al. (2020) showed that the genomic regions associated with traits of interests could be detected by the same approach, and our results in SCEN5 also showed a higher power for QTL detection under lower nQTL. For the accuracy of GEBV, Takeda et al. (2020) obtained the realized reliability as the indicator of the accuracy of GEBV using de‐regressed EBV as true value. Taking the square root of the coefficient of determination of regression of de‐regressed EBV as the GEBV, the values (regarded as accuracy) ranged from 0.3 to 0.5, being approximately equivalent to the results of the current study in SCEN5 with 0.41 to 0.49. This suggests that the current simulation process is appropriate, but the accuracies of GEBV in real data and the simulated population using genotyped bulls are limited, even if we determined that the genotypes of bulls with several phenotyped progenies were valuable to the prediction of GEBV in this study. If there were many individuals with phenotypic data, obtaining the genotypes of the bulls could allow us to accurately determine the QTL regions. However, to increase the accuracy of GEBV, it is necessary to enhance the reference population consisting of individuals with both genotypes and phenotypes. Over 4,500 individuals are needed to obtain a high accuracy of 0.7, such as SCEN2 used in this study.

## 5 CONCLUSION

We evaluated the effects of genotyped bulls with a different number of progenies on the power of QTL detection and the accuracy of GEBV. We found that a small number of genotyped bulls could be available for QTL detection when the bulls have a large number of phenotyped progeny and the genetic background of the target trait includes some major QTLs. Therefore, cost‐effective QTL detection could be performed depending on these conditions. Additionally, using a reference population, including the genotypes of bulls that have more progenies with phenotypes, enabled more accurately predicted GEBV. However, if practical use of genomic evaluation, such as the selection of sire candidates is considered, more than 4,500 animals with both genotypes and phenotypes would be required.

## CONFLICT OF INTEREST

The authors declare no conflicts of interest associated with this manuscript.

## Supporting information

Figure S1

Figure S2
