# Supplementary material for: Effect of genotyped bulls with different numbers of phenotyped progenies on quantitative trait loci detection and genomic evaluation in a simulated cattle population
Source: Anim Sci J. 2020 Aug 11;91(1):e13432. doi: 10.1111/asj.13432 (PMC7507195; doi:10.1111/asj.13432)
Supplement: Supplementary file 1 — Figure S1 [file ASJ-91-e13432-s001.pdf]

(a)

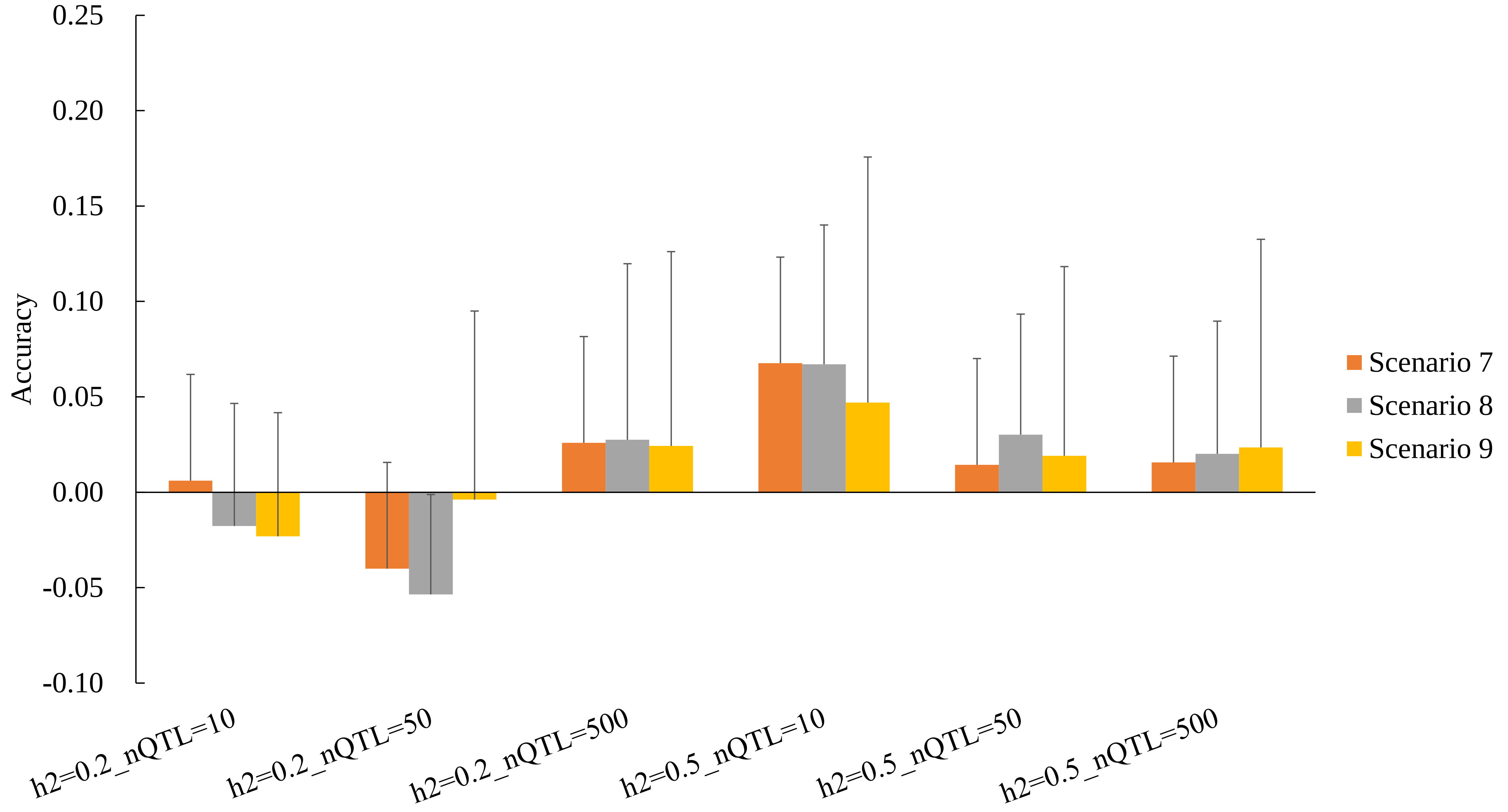

(b)

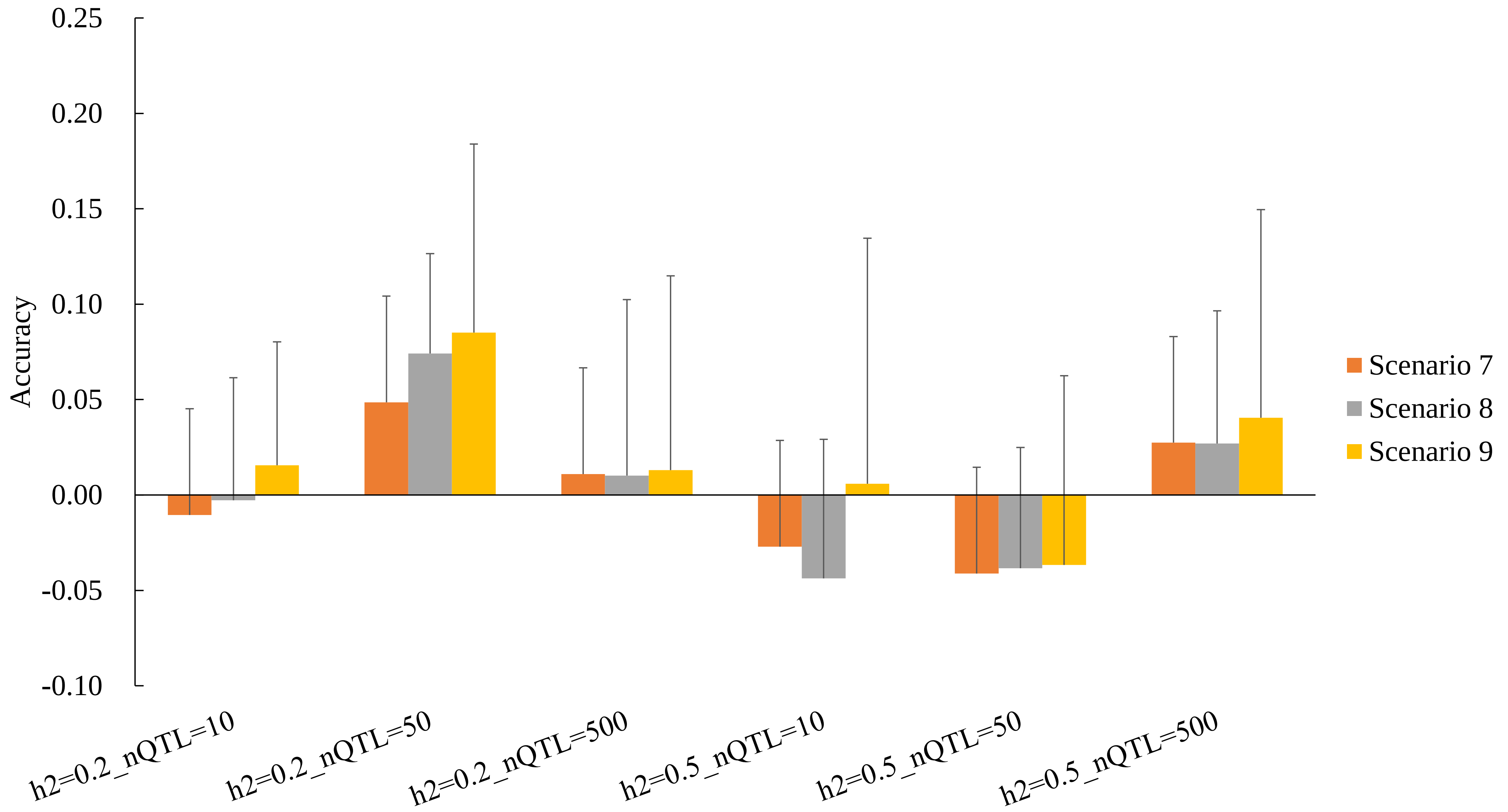

Figure S1. The accuracy of estimated breeding values in the scenarios. (a), Ne of 20; (b), Ne of 100; h2, heritability; quantitative trait loci (QTL); nQTL, number of QTL. The y-axis indicates the accuracy of estimated breeding values.
