# Supplementary material for: Effect of genotyped bulls with different numbers of phenotyped progenies on quantitative trait loci detection and genomic evaluation in a simulated cattle population
Source: Anim Sci J. 2020 Aug 11;91(1):e13432. doi: 10.1111/asj.13432 (PMC7507195; doi:10.1111/asj.13432)
Supplement: Supplementary file 2 — Figure S2 [file ASJ-91-e13432-s002.pdf]

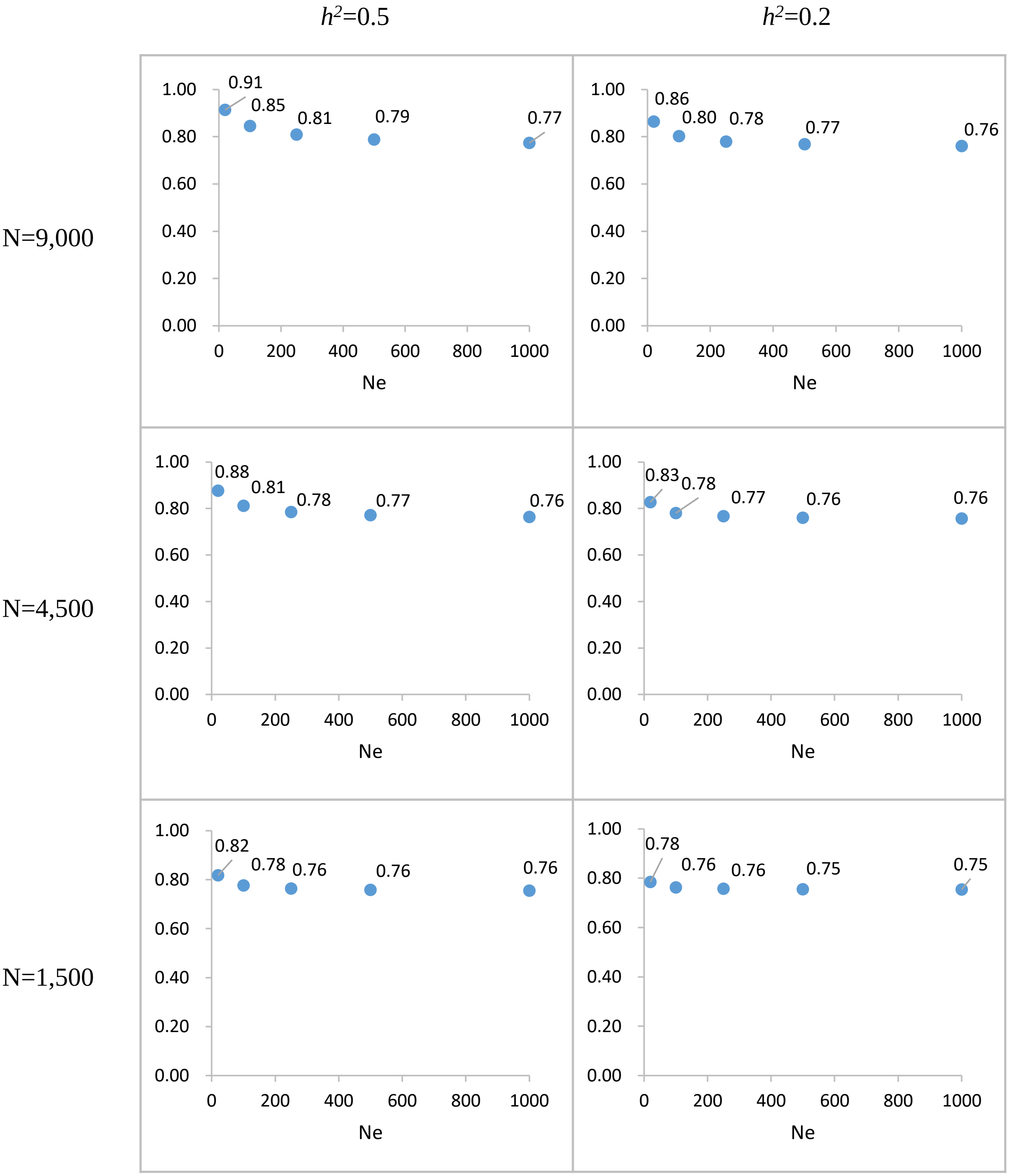

Figure S2. Accuracy of genomic estimated breeding values (GEBVs) depending on the effective population size (Ne = 20, 100, 250, 500, or 1,000) calculated with the formula of Goddard (2009) using number of individuals with phenotypic records (N = 9,000, 4,500 or 1,500), heritability (h^2 = 0.2 or 0.5), and length of the chromosome (= 30). After calculating the reliability of GEBV using the formula, the square root was used to determine the accuracy of GEBV. In each graph, the y-axis indicates the accuracy of GEBVs and x-axis indicates the Ne.
